# Mock Samples That Mimic Human Cervicovaginal Samples to Accelerate the Development and Evaluation of Assays for High‐Risk HPV for Cervical Cancer Screening

**DOI:** 10.1002/jmv.70931

**Published:** 2026-04-16

**Authors:** Emilie Newsham Novak, Ariel Ma, Cannon J. Hansen, Zoha Wazir, Meaghan Bond, Michael E. Scheurer, Ming Guo, Mila P. Salcedo, Kathleen M. Schmeler, Rebecca R. Richards‐Kortum

**Affiliations:** ^1^ Department of Bioengineering Rice University Houston TX USA; ^2^ Department of Pediatrics, Division of Hematology/Oncology Emory University School of Medicine Atlanta GA USA; ^3^ Department of Anatomical Pathology The University of Texas MD Anderson Cancer Center Houston TX USA; ^4^ Department of Gynecologic Oncology and Reproductive Medicine The University of Texas MD Anderson Cancer Center Houston TX USA

## Abstract

Nearly all cervical cancer cases are caused by high‐risk human papillomavirus (hrHPV) infections. The World Health Organization recommends screening for hrHPV using nucleic acid amplification tests (NAATs) that detect hrHPV DNA or mRNA. Lack of access to affordable, point‐of‐care screening tests in resource‐limited settings leads to women presenting with advanced‐stage cervical cancer, with many dying of the disease. There is a significant need to develop point‐of‐care NAATs to improve screening and early detection. Because access to real clinical samples is limited, initial evaluation of NAATs is often performed using contrived samples created using some combination of extracted hrHPV DNA and/or cultured hrHPV‐positive cells. When mock samples do not adequately recapitulate the contents of clinical cervicovaginal samples, it can delay clinical translation of potentially promising assays. To improve the value of contrived samples, we characterized the composition of 32 hrHPV DNA‐positive cervicovaginal clinical samples. We also describe a simple method to generate contrived samples that mimic the diversity of clinical cervicovaginal samples, and test them using the methods used to characterize the clinical samples. Results show that the hrHPV DNA content of cervicovaginal samples varies by approximately eight orders of magnitude and spans from 100% linear integrated DNA to 100% circular non‐integrated DNA, that the concentration of hrHPV mRNA also varies by nearly nine orders of magnitude between patient samples, and that the concentration of potential inhibitors such as hemoglobin varies by more than three orders of magnitude. hrHPV DNA and mRNA extracted from contrived samples exhibited expected patterns of DNA quantity, DNA conformation, and mRNA quantity. Altogether, the protocols described here to generate mock samples can help NAAT developers optimize test performance prior to clinical evaluation, potentially improving test performance and reducing time to deployment.

## 1 Introduction

Globally, cervical cancer is the fourth most common cancer in women, with approximately 660,000 new cases and 350,000 deaths in 2022 [1]. Nearly all cervical cancer cases are caused by persistent infection with high‐risk types of human papillomavirus (hrHPV), a DNA virus. Most hrHPV infections clear naturally, but some persistent infections result in integration of viral DNA into the host cell genome, leading to precancerous cellular changes. Left untreated, precancerous lesions may progress to cervical cancer over years to decades [2]. This slow progression affords many opportunities to prevent cervical cancer through vaccination, screening to identify at‐risk patients, and excision or ablation of precancerous lesions [3]. These interventions require infrastructure that is not available in many low‐resource settings (LRS), making the burden of cervical cancer disproportionately high in LRS globally – approximately 90% of cervical cancer deaths occur in low and middle‐income countries [4, 5].

The World Health Organization (WHO) recommends screening for hrHPV using nucleic acid amplification tests (NAATs) [6]. hrHPV DNA is detectable in patients with active hrHPV infections, making it a sensitive (> 90%) biomarker for high‐grade cervical precancer or cancer [7]. Polymerase chain reaction (PCR)‐based tests are the gold standard for hrHPV DNA detection, and commercially available tests include cobas (Roche), Onclarity (BD), RealTime (Abbott), and Xpert HPV (Cepheid) [8]. Compared to other options, Xpert is fast and easy to use, providing sample‐to‐answer detection of 14 hrHPV types in less than 1 h [9]. This makes it compatible for screen‐and‐treat cervical cancer prevention programs in LRS, where patients are screened for hrHPV and treated for cervical precancer in a single visit to reduce loss to follow‐up [6]. hrHPV DNA tests suffer from a lack of specificity, with a positive predictive value (PPV) of only 15‐25% for high‐grade cervical precancer, as many hrHPV infections spontaneously clear. For this reason, hrHPV E6/E7 mRNA, which is over‐expressed from viral DNA following integration, is a more specific biomarker for high‐grade cervical precancer [10]. There are some commercially available hrHPV mRNA NAATs, including Aptima (Hologic) and HPV‐Proofer (PreTect) [8]. These selected tests, their limits of detection, and whether they have an internal cellular control are summarized in Table S1 [8, 11, 12, 13, 14, 15, 16].

None of these commercially available tests meet the WHO REASSURED criteria for diagnostic test accessibility in LRS [17]. Xpert HPV, for example, is prohibitively expensive, even with global access pricing, and requires a stable electrical connection and operation under precise temperature conditions, making it inaccessible and unusable in many LRS [18, 19]. There is a critical need for point‐of‐care hrHPV NAATs that meet REASSURED criteria. Recent literature describes prototypes of hrHPV DNA and mRNA tests that could be performed in LRS [20, 21, 22, 23, 24, 25, 26]. Most reports of test development include efforts to optimize performance using synthetic samples of increasing complexity, followed by clinical testing with extracted nucleic acids. For DNA test development, experimental samples range from short single‐stranded sequences of the genetic target (gBlocks) in nuclease‐free water to full‐length plasmid DNA or DNA extracted from hrHPV‐positive cell lines or cervicovaginal samples [23, 27]. For mRNA tests, experimental samples include *in vitro* transcribed mRNA or extracted mRNA from hrHPV‐positive cell lines or cervicovaginal samples [25, 28]. Because DNA or RNA extraction is not compatible with point‐of‐care testing, none of these samples are good analogs to evaluate and optimize a sample‐to‐answer test workflow [29].

Extraction‐free sample preparation can be evaluated with cultured hrHPV‐positive cell lines, but cell monocultures fail to recapitulate the complex cellular properties and sample matrix of cervicovaginal samples. While available for purchase and easy to grow and maintain, hrHPV‐positive cells are insufficient cervicovaginal sample models for NAAT development for many reasons. First, not all cells in an hrHPV‐positive clinical sample contain integrated viral DNA, which markedly affects the DNA/RNA content in the sample [30]. Second, not all viral DNA will be integrated and therefore linear; some will still be in its non‐integrated episomal (circular) form [31]. This affects the ability of enzymes associated with sample preparation and nucleic acid amplification to interact with viral DNA. Third, hrHPV DNA and mRNA content vary depending on stage of infection, level of progression, and HPV type [32, 33]. Finally, cervicovaginal samples contain many potential biochemical inhibitors, including blood, mucus, off‐target viruses and bacteria, and the cell‐preserving collection media. The method of sampling (provider vs. self‐collection, brush type, etc.) also impacts the sample matrix and DNA/RNA content [34, 35, 36]. It is also important to consider that the inappropriate use of lubricants during sample collection can impact sample evaluation [37].

Ideally, freshly collected cervicovaginal samples would be used, but this requires access to clinical samples and is often infeasible in the assay development stage. It is also difficult to compare different tests if they are optimized and evaluated with different clinical samples. As such, there is a need for a mock cervicovaginal sample that recapitulates patient cervicovaginal samples to facilitate rapid development of translatable point‐of‐care hrHPV NAATs.

In this study, we characterize 32 freshly collected cervicovaginal samples for total cell count, hrHPV DNA quantity, hrHPV DNA conformation (integrated linear or non‐integrated circular), hrHPV E7 mRNA quantity, and hemoglobin content. Using this information, we provide a recipe to create mock cervicovaginal samples that more accurately mimic the properties of freshly collected clinical samples.

## 2 Methods

### 2.1 Cervicovaginal Sample Collection

Cervicovaginal samples were collected from patients referred to the colposcopy clinic at The University of Texas MD Anderson Cancer Center and Lyndon B. Johnson Hospital. Women were eligible to participate if they (1) had a cervix, (2) were 21 years of age or older, (3) were scheduled to undergo hrHPV testing according to national or institutional guidelines at time of enrollment and/or were scheduled to undergo biopsy, loop electrosurgical excision procedure (LEEP), and/or or endocervical curettage (ECC), (4) were willing and able to provide written informed consent, (5) were able to perform protocol‐required activities, and (6) were able to speak and read English or Spanish. This study was reviewed and approved by the Institutional Review Boards at The UT MD Anderson Cancer Center, Lyndon B. Johnson Hospital, and Rice University under IRB protocols 2024‐0020 (The UT MD Anderson Cancer Center and Lyndon B. Johnson Hospital) and FY2024‐388 (Rice University).

Fixatives based on ethanol and methanol facilitate both the preservation and extraction of DNA. In this study, we utilized PreservCyt, a methanol‐based fixative commonly employed in clinical settings [38]. For patients scheduled to undergo hrHPV testing as part of their standard‐of‐care evaluation, a cervicovaginal sample was first collected by a provider into PreservCyt buffer. A subsequent cervicovaginal sample was collected by a provider into PreservCyt for all patients. During sample collection, a minimal amount of water‐soluble, carbomer‐free lubricant was applied only to the outer tip of the speculum blades, avoiding contact with the cervical os or vaginal walls. No lubricant was applied to the sampling device.

The standard‐of‐care sample was used for clinical hrHPV testing with cobas 4800 (Roche Diagnostics) or Aptima (Hologic). The subsequent cervicovaginal sample collected for all patients was placed for immediate testing with the GeneXpert HPV test (Cepheid). The LOD for each hrHPV genotype for each NAAT used in the clinical sample evaluation is listed in Table S2 [12, 39, 40]. For patients scheduled to undergo biopsies, loop electrosurgical excision procedures (LEEPs), or endocervical curettages (ECCs), tissue samples were submitted for routine histopathologic analysis and diagnosis.

### 2.2 Sample Processing

32 provider‐collected cervicovaginal samples that were positive for hrHPV on the Xpert HPV test were assessed for DNA, mRNA, and blood content, as described below. To extract total DNA and RNA, 1 mL of sample was centrifuged at 5000 × g for 10 min and the supernatant was aspirated. DNA was extracted from the pellet using the Monarch Genomic DNA (gDNA) Purification Kit (New England Biolabs) and eluted into 100 µL nuclease‐free water. To digest linear DNA while leaving circular, non‐integrated DNA intact, extracted DNA was treated with Exonuclease V (RecBCD) (New England Biolabs) using methods described by Izadi and colleagues [31].

RNA was extracted using the Monarch Total RNA Miniprep Kit (New England Biolabs) and eluted into 100 µL DEPC‐treated water and split into two 50 µL aliquots. Each aliquot was incubated with 1.5 µL DNase I (RNase‐free), 15 µL DNase I reaction buffer (New England Biolabs), and 83.5 µL DEPC‐treated water in a nonstick RNase‐free microfuge tube at 37°C for 2 h. Then, the Monarch RNA Cleanup Kit (New England Biolabs) was used to remove the DNase with final elution into 50 µL DEPC‐treated water. EDTA (Thermo Fisher Scientific) was added to 2 mM final concentration to preserve RNA.

### 2.3 DNA and RNA Quantification

Genomic and hrHPV DNA were quantified from extracted total DNA using quantitative polymerase chain reaction (qPCR) with SYBR intercalating dye and primer sequences for the HPV 16, 18, and 45 E7 gene and the human β‐actin gene, as described previously [27]. HPV standard curves were generated with E7 HPV gBlocks quantified with ATCC or WHO quantitative standards. Genomic DNA standard curves were generated using genomic DNA from human Jurkat cells (Thermo Fisher Scientific), quantified with a nanodrop spectrophotometer.

HPV and β‐actin mRNA were quantified from extracted total RNA using reverse transcription qPCR (RT‐qPCR). HPV standard curves were generated with hrHPV mRNA previously quantified with E7 gBlocks, assuming 100% transcription efficiency. β‐actin mRNA was quantified using Jurkat genomic DNA as a standard, quantified with a nanodrop spectrophotometer. Table S3, Table S4, and Figure S1 provide an example of the experimental data and standard curve used to quantify hrHPV mRNA in a clinical sample. A standard curve generated from a known number of Jurkat DNA β‐actin copies was used to quantify the number of cells in clinical samples. Episomal DNA was quantified by using the extracted DNA treated with Exonuclease V in the qPCR assays for HPV 16, HPV 18, and HPV 45. A Kruskal‐Wallis test was used to compare the copies of DNA and the percent of circular DNA per cell between samples positive for HPV 16, HPV 18, and HPV 45.

Melt curve analyses were performed for both qPCR and RT‐qPCR reactions to differentiate on‐target from off‐target amplification due to the use of nonspecific intercalating dye in these reactions.

To quantify mRNA clinical samples, RNA and DNA were extracted from equal volumes of well‐mixed clinical samples and quantified with RT‐qPCR and PCR, respectively. The copies of hrHPV mRNA per cell were estimated by dividing the mRNA quantity by one‐half the copies of gDNA detected from the same volume of sample. A Mann–Whitney *U*‐test was performed to assess the difference in medians between samples with corresponding pathology less than CIN2 and greater than or equal to CIN2.

All statistical analyses were performed using GraphPad Prism 10.

### 2.4 Hemoglobin Quantification and pH Measurement

The amount of hemoglobin present in cervicovaginal samples was measured because it inhibits DNA polymerase, a key component of most amplification reactions [41]. Sample absorbance was measured and used to quantify hemoglobin. PreservCyt media contains methanol; accordingly, hemoglobin concentration was calculated based on the absorbance of methemoglobin [42]. A standard curve was generated using whole blood from a volunteer with hemoglobin measured using a COULTER A^C^•T diff 2 Analyzer. Whole blood was diluted in PreservCyt to 0.0614 g/dL, 0.0082 g/dL, 0.00328 g/dL, and 0.00164 g/dL total hemoglobin concentration by volume. Aliquots were refrigerated at 4°C for 7 days to reflect similar time and storage conditions to patient cervicovaginal samples. This incubation permitted full conversion of hemoglobin to methemoglobin.

Following incubation, each dilution was added to a cuvette (UVette 220–1600 nm, 50–2000 µL; Eppendorf AG). Two additional cuvettes were filled with 500 µL of PreservCyt for baseline measurements. Absorbance was measured between 350 nm and 800 nm using a spectrophotometer (Cary 5000 UV‐Vis‐NIR Spectrophotometer, Agilent Technologies) with baseline correction. The baseline was calibrated with a 500 µL sample of PreservCyt media; this value was used for the baseline correction of all other measurements. To correct for turbidity, absorbance at 800 nm was subtracted from the 404 nm absorbance value. The corrected absorbance at 404 nm was used to generate a line of best fit with a y‐intercept of zero.

500 µL of each hrHPV DNA‐positive cervicovaginal sample in PreservCyt was added to a cuvette and measured as described above; the standard curve was used to estimate blood content. For samples where absorbance exceeded 2.5, the sample was diluted 10‐fold, and the calculated value was multiplied by ten.

The pH of thirteen cervicovaginal samples (Samples 5 and 21–32) was measured in triplicate using a Mettler Toledo LE422 pH meter.

### 2.5 Cell Culture

HeLa (HPV 18‐positive), SiHa (HPV 16‐positive), MS751 (HPV 45‐positive), and CaSki (HPV 16‐positive) cells (ATCC) were cultured according to ATCC recommendations. Cells were harvested immediately prior to use with Trypsin‐EDTA and resuspended in phosphate‐buffered saline.

To quantify mRNA copies per cell for cell lines, harvested cells were split into two equal aliquots, and DNA and RNA were extracted from each. The amount of hrHPV mRNA copies per cell was estimated as described above.

### 2.6 Mock Sample Generation and Evaluation

In creating mock samples to mimic the properties of clinical samples relevant for the development and evaluation of NAATs, we began with pooled hrHPV‐negative cervicovaginal samples. A background of pooled negative samples was used to more accurately represent the sample matrix found in a patient's cervicovaginal sample, including HPV‐negative squamous cervical cells, potential biochemical inhibitors such as blood, mucus, off‐target viruses and bacteria, and the cell‐preserving collection media. The pooled negative sample was created by mixing 2 mL each from five samples confirmed hrHPV DNA and RNA‐negative in PreservCyt for a total of 10 mL. To mimic the presence of hrHPV infection, we then considered the addition of the following possible ingredients: cultured hrHPV‐positive cervical cells, which contain linear, integrated hrHPV DNA and hrHPV mRNA; full genome hrHPV plasmids, which are double‐stranded circular DNA, to mimic the presence of HPV virus in episomal form; cell‐free mRNA extracted from hrHPV‐positive cells; and hemoglobin, a potential interferant. Three types of mock samples were generated in a pooled negative cervicovaginal sample to mimic three distinct clinical samples. Each mock sample mimicked a clinical sample collected and evaluated previously as closely as possible, including its HPV type. The proportion of ingredients was adjusted to ensure that DNA and RNA quantities spanned the range observed in patient samples.

As described in Table 1, a “No Integration” mock sample with 0% integrated DNA was developed to mimic the properties of an HPV 45 DNA‐positive, HPV mRNA‐negative sample. The No Integration mock sample was created by adding a full genome HPV 45 plasmid (courtesy of the CDC) to pooled negative media to a final concentration of 100,000 copies per mL. A “Partial Integration” mock sample was developed to mimic an HPV 18‐DNA and HPV 18 mRNA positive sample with approximately 50% integrated and 50% non‐integrated HPV 18 DNA. The Partial Integration mock sample was created by adding HeLa cells to a final concentration of 150,000 cells per mL and full genome HPV 18 plasmid (courtesy of the CDC) to a final concentration of 3,000,000 copies per mL. The “Full Integration” mock sample was developed to mimic an HPV 16 DNA and HPV 16 mRNA‐positive sample with 100% integrated DNA. The Full Integration mock sample was generated by adding SiHa cells to a final concentration of 50,000 cells per mL.

**Table 1:** hrHPV‐positive mock sample components and recommended concentrations.

Component	Recommended concentrations	Possible sources
Blood (hemoglobin)	High: > 0.01 g/dL	1. Whole blood from normal volunteers is available for purchase 2. Human hemoglobin powder available for purchase
	Medium: 0.001–0.01 g/dL	
	Low: 0–0.001 g/dL	
HPV DNA: episomal and/or integrated	High: > 100 cp per total cells in sample	1. Whole cultured hrHPV‐positive cells (integrated) 2. Extracted DNA from hrHPV‐positive cell lines (integrated) 3. Full genome hrHPV plasmids (episomal)
	Medium: 1–100 cp per total cells in sample	
	Low: < 1 cp per total cells in sample	
HPV mRNA	High: > 10 cp per total cells in sample	1. HPV‐positive cell lines 2. Extracted RNA from hrHPV‐positive cell lines 3. *In vitro* transcribed mRNA
	Medium: 0.1–10 cp per total cells in sample	
	Low: < 0.1 cp per total cells in sample	

Each mock sample was evaluated for hrHPV DNA quantity and conformation and hrHPV mRNA quantity as described for the patient cervicovaginal samples, with slight modifications. The purpose of this characterization was to demonstrate that mock samples can withstand standard sample preparation methods that might be required for NAAT development. Each well‐mixed mock sample was split into two 500 µL aliquots for DNA and RNA extraction, respectively. Each aliquot was centrifuged at 5000 × g for 10 min; the supernatant and cell pellet were then processed separately using the methods described above, with the exception that the lysis step in both the DNA and RNA kits was not performed for the supernatant. The final DNA and RNA extractions were eluted into 25 µL for each sample component and combined to a final volume of 50 µL, representing a 10X concentration, which is equal to the concentration at which patient samples were processed.

DNA and RNA from the mock samples were quantified using qPCR and RT‐qPCR as described above. Treatment with Exonuclease V was also performed on extracted DNA to quantify the amount of integrated and episomal DNA remaining after extraction. Each of the mock samples was also tested using the Xpert HPV test, and the hemoglobin concentration was calculated for each mock sample using the methods described above.

**Figure 1:**
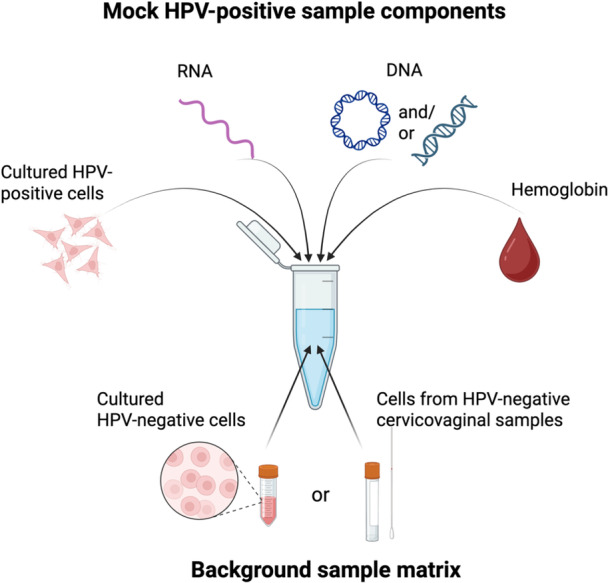
Components recommended to generate an hrHPV‐positive mock cervicovaginal sample. The background sample matrix can be composed of either hrHPV‐negative human cervicovaginal samples or cultured hrHPV‐negative cervical cells spiked into a representative background of the sample collection matrix. Additional components to mimic an hrHPV‐positive sample include hemoglobin, DNA, RNA, and/or cultured hrHPV‐positive cell lines.

Figure 1 displays the components recommended to generate mock hrHPV‐positive cervicovaginal samples. The background sample matrix should represent the sample and collection media as accurately as possible. If banked negative cervicovaginal samples in the sample collection medium are unavailable, they can be generated from purchased or cervicovaginal swabs collected from normal volunteers (with appropriate testing to confirm they are hrHPV‐negative) [43]. Alternatively, cultured hrHPV‐negative cell lines can be added to the sample collection media with pooled vaginal fluid to represent a cervicovaginal swab. The components to be added to the background sample matrix and the range of concentrations to generate different physiologically representative mock hrHPV‐positive cervicovaginal samples are listed in Table 1. The HPV copies per total cells in the sample are a ratio of HPV copies to the total (both HPV‐positive and HPV‐negative) cervical cells in the sample.

## 3 Results

### 3.1 HPV DNA Quantity and Conformation in hrHPV DNA‐Positive Cervicovaginal Samples

**Figure 2:**
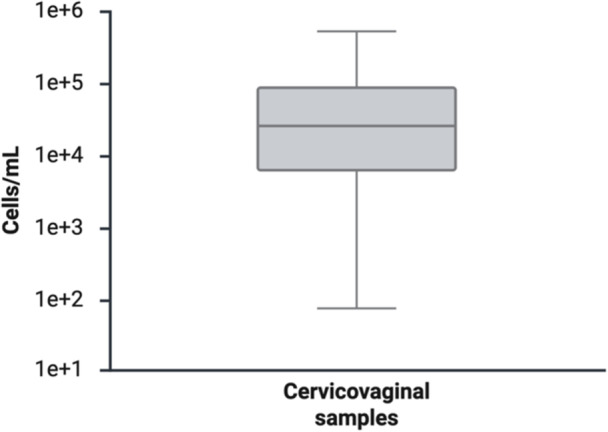
Concentration (cells/mL) of 32 cervicovaginal samples.

**Figure 3:**
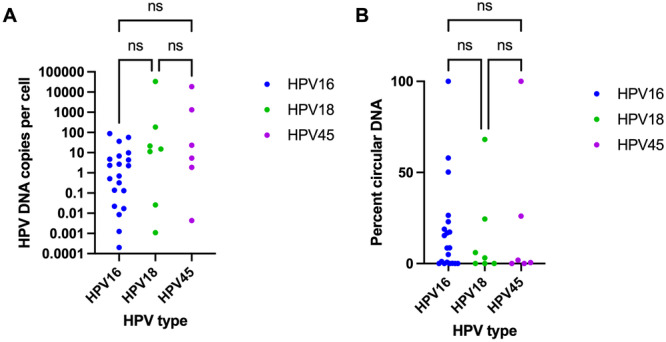
hrHPV DNA quantity and conformation in 32 HPV DNA‐positive cervicovaginal samples. (A) Number of HPV 16, HPV 18, and HPV 45 E7 DNA copies per cell in HPV 16, HPV 18, and HPV 45‐positive samples. (B) Percent of circular (non‐integrated) hrHPV DNA out of total hrHPV DNA in HPV 16, HPV 18, and HPV 45‐positive samples.

The 32 hrHPV‐positive cervicovaginal samples had a median concentration of approximately 42,000 cells/mL (Figure 2), with a range consistent with other studies in which the same collection method and media were used [44]. The amount of hrHPV DNA per cell in 32 hrHPV‐positive cervicovaginal samples varied by eight orders of magnitude (Figure 3A), from approximately 0.001 copies per cell to more than 10,000 copies per cell. The amount of hrHPV DNA per 20 mL original sample is shown in Figure S2. There was no statistically significant difference between the number of DNA copies per cell (*p* = 0.17) or percent of circular hrHPV DNA (*p* = 0.82) for samples positive for HPV 16, HPV 18, and HPV 45. The fraction of non‐integrated (circular) hrHPV DNA varied from 100%, indicating that all hrHPV DNA in the sample was in its circular, episomal form, to 0%, indicating that the sample contained entirely linear, integrated DNA (Figure 3B).

### 3.2 hrHPV mRNA Quantification in hrHPV DNA‐Positive Cervicovaginal Samples

**Figure 4:**
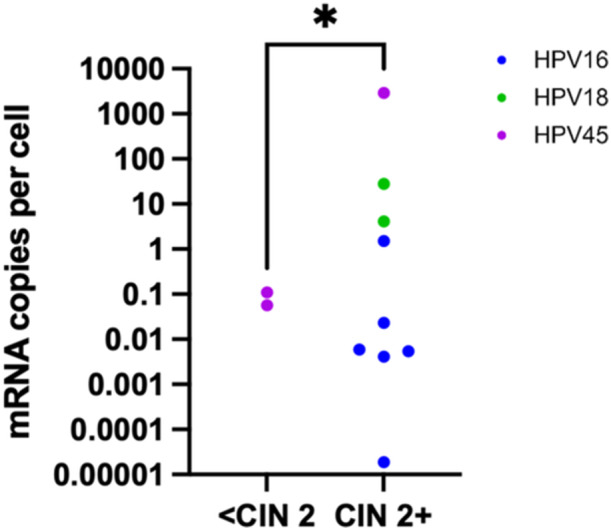
hrHPV mRNA in 11 hrHPV‐DNA positive cervicovaginal samples. HPV mRNA copies per cell stratified by pathology <V/VA/CIN 2 or V/VA/CIN 2 +.

Of the 32 hrHPV‐DNA positive cervicovaginal samples, eleven had detectable hrHPV mRNA. The number of copies of hrHPV mRNA per cell varied by nearly nine orders of magnitude across the samples (Figure 4). Pathology from cervical, vaginal, or vulvar biopsies taken at the same time as sample collection was available for six samples. Two samples did not have corresponding biopsies. Overall, the samples with vulvar/vaginal/cervical intraepithelial neoplasia 2 or higher (V/VA//CIN 2+ or adenocarcinoma in situ (AIS)) pathology have more copies of hrHPV mRNA per cell than the sample with CIN 1 pathology, according to a Mann–Whitney *U*‐test comparing the medians of the two sample sets (*p* = 0.04).

### 3.3 Blood Content and pH of Cervicovaginal Samples

**Figure 5:**
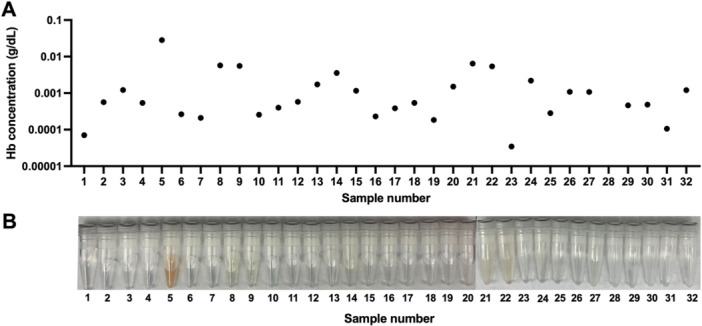
Hemoglobin concentration in cervicovaginal samples. (A) Hemoglobin (Hb) concentration (g/dL) in 32 HPV DNA‐positive cervicovaginal samples. (B) Photo of 500 µL of each sample.

Among the 32 HPV‐positive cervicovaginal samples, the hemoglobin concentration ranged from 0 to 28 mg/dL (Figure 5). Among a thirteen‐sample subset of HPV‐positive cervicovaginal samples, the mean pH fell between 5.7 and 6.1 (Figure S3).

All clinical sample results are summarized in Table S6.

### 3.4 HPV DNA and mRNA Quantification in Established Cell Lines

**Figure 6:**
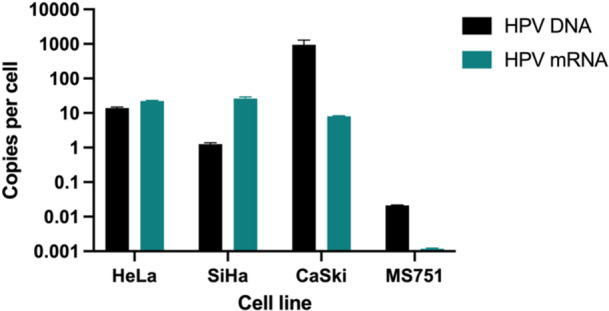
hrHPV DNA and mRNA quantity in cell lines. hrHPV DNA and mRNA copies per cell in HeLa, SiHa, CaSki, and MS751 cultured cells. Error bars represent standard deviation.

The amount of hrHPV mRNA expressed per cell varies between HeLa, SiHa, MS751, and CaSki cell lines (Figure 6). While HeLa, SiHa, and CaSki all express hrHPV mRNA on the same order of magnitude, MS751 expresses significantly less hrHPV mRNA per cell. The amount of hrHPV mRNA compared to the amount of hrHPV DNA varies between the different cell lines. Results of quantification of copies of hrHPV DNA per cell agreed with established literature for HeLa, SiHa, and CaSki cells (Table 2). While HeLa expressed approximately the same order of magnitude of mRNA copies per cell as DNA, SiHa expressed more hrHPV mRNA copies than hrHPV DNA per cell, and CaSki and MS751 both had approximately two orders of magnitude more hrHPV DNA than hrHPV mRNA per cell.

**Table 2:** hrHPV DNA and mRNA copies per cell from the literature and calculated experimentally.

Cell line (HPV type)	HeLa (HPV 18)	SiHa (HPV 16)	CaSki (HPV 16)	MS751(HPV 45)
HPV DNA copies per cell (literature) [45]	~20–50	~1–2	~600	~1
HPV DNA copies per cell (experimental)	14.0	1.2	907.7	0.02
HPV mRNA copies per cell (experimental)	22.3	26.1	8.0	0.001

### 3.5 Mock Sample Evaluation

Mock samples were created according to Table 3 and evaluated for DNA content (quantity and conformation) and mRNA content to demonstrate the ability of the mock samples to mimic patient samples throughout standard sample preparation processes. They were also tested using the Xpert HPV test to confirm their functionality with a commercially available hrHPV test (Table S4).

**Table 3:** Protocol for generating each mock sample.

Mock sample	Clinical sample represented	Components (concentrations)
No integration	Sample 2	1. Sample background of five pooled hrHPV‐negative cervicovaginal samples in PreservCyt 2. Full genome HPV 45 plasmid (100,000 cp/mL)
Partial integration	Sample 9	1. Sample background of five pooled HPV‐negative cervicovaginal samples in PreservCyt 2. Cultured HeLa cells (150,000 cells/mL) 3. Full genome HPV 18 plasmid (3,000,000 cp/mL)
Full integration	Sample 5	1. Sample background of five pooled hrHPV‐negative cervicovaginal samples in PreservCyt 2. Cultured SiHa cells (50,000 cells/mL)

**Figure 7:**
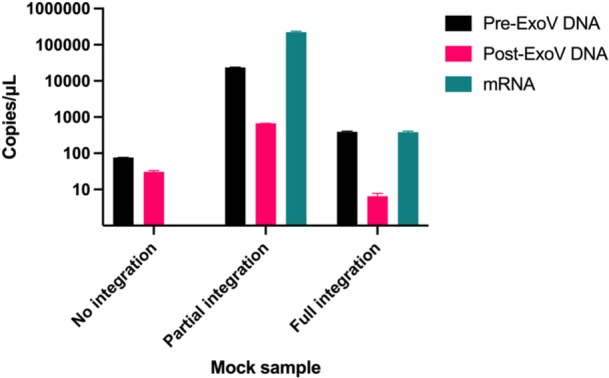
Evaluation of mock samples. hrHPV DNA copies per µL before treatment with Exonuclease V (Pre‐ExoV), after treatment with Exonuclease V (Post‐ExoV), and hrHPV mRNA copies per µL for the No Integration, Partial Integration, and Full Integration mock samples. Error bars represent standard deviation.

The No Integration mock sample performed as expected (Figure 7): since only plasmid (circular) HPV 45 DNA was added to the pooled negative sample, the amount of DNA remaining after treatment with linear DNA‐specific Exonuclease V was on the same order of magnitude as the original extracted DNA, and there was no HPV 45 E7 mRNA. The Partial Integration mock sample, which contained approximately equal amounts of integrated and non‐integrated HPV 18 E7 DNA, performed approximately as expected, with more DNA prior to Exonuclease V treatment than after, and the large amounts of detectable mRNA expected from HeLa cells. Theoretically, the quantity of DNA following Exonuclease V treatment should have been approximately 12,000 copies per µL; in practice, it was approximately 700 copies per µL, indicating degradation of approximately an order of magnitude. The Full Integration mock sample, which contained only integrated linear HPV 16 E7 DNA in the form of SiHa, behaved as expected, with most DNA degraded by Exonuclease V and large amounts of detectable HPV 16 E7 mRNA. Of note, each type of mock sample in Table 3 (No Integration, Partial Integration, and Full Integration) could be generated with cells and plasmids corresponding to any hrHPV genotype.

The Xpert HPV test accurately detected HPV 18/45 in the No Integration sample, HPV 18/45 in the Partial Integration sample, HPV 16 in the Full Integration sample, and no HPV in the hrHPV‐negative pooled mock sample background (Table S4). As shown in Table S5, the hemoglobin concentration in the mock samples (resulting from the hemoglobin in the pooled hrHPV‐negative sample) fell within the hemoglobin range of the 32 clinical samples.

## 4 Discussion

Developing NAATs to make hrHPV testing more accessible is an important endeavor, but NAATs must be developed and optimized using samples that represent the test use case. When a sample bank is unavailable, researchers should optimize tests using synthetic samples that recapitulate clinical samples as closely as possible.

The evaluations described here demonstrate that there is large biological diversity in DNA quantity, DNA conformation, mRNA quantity, and blood content among 32 hrHPV DNA‐positive cervicovaginal samples. This observation highlights that it is not sufficient to design an HRHPV NAAT for the smallest amount of extracted target, as is often described in literature; rather, tests must accommodate the full extent of possible sample contents. This holds especially true for point‐of‐care tests, in which sample pre‐processing is minimal.

The different conformations and concentrations of hrHPV RNA and DNA in cervicovaginal samples have significant implications for NAAT development. First, a clinically relevant test for hrHPV DNA must have a wide dynamic range to encompass the range of possible concentrations within a sample. The amount of hrHPV DNA in a sample is positively correlated with the risk for cervical cancer [46]. Due to the natural history of cervical cancer, there is also a relationship between cervical cancer risk and the amount of integrated hrHPV DNA versus circular, non‐integrated DNA [47]. The conformation of DNA may also have implications for point‐of‐care hrHPV DNA and mRNA test development, as the DNA conformation affects the efficiency of sample preparation techniques, such as nuclease treatment for the removal of both forms of DNA while maintaining mRNA integrity [48]. The replication of circular DNA has also been demonstrated to be less efficient than linear DNA in qPCR [49].

Mock samples were generated to provide researchers with a protocol to create samples that more accurately recapitulate patient samples. Ideally, pooled hrHPV‐negative cervicovaginal samples are used to create mock samples. If pooled negative samples are not available, dry vaginal swabs or pooled vaginal fluid can be purchased from many vendors. Vaginal samples, however, may not contain as many exfoliated cervical cells as a cervicovaginal sample. In this instance, an hrHPV‐negative cervical cell line, such as C33A, could be added to the appropriate proportion with hrHPV‐positive cervical cells to mimic the disease grade of interest. In high‐grade cervical precancer, for example, approximately 8–18% of cells express high levels of hrHPV E6/E7 mRNA, indicating integrated viral DNA [30].

There are a few key limitations of this study. First, the immortalized cells used here to represent hrHPV‐infected cells (HeLa, SiHa, and MS751) are limited in their representation of patient samples, but in the absence of clinical samples for diagnostic assay validation, they are an accessible bridge to clinical samples. Immortalized cells are less costly and easier to maintain than primary cells. For this reason, it is important that immortalized cell lines be used in combination with as representative a sample background as possible, such as the pooled HPV‐negative cervicovaginal samples used here. If available, direct validation with clinical samples is preferable to mock samples. Second, the inclusion of only three hrHPV genotypes does not represent the full spectrum of high‐risk HPVs – HPV 16, HPV 18, and HPV 45 are responsible for only about 75% of cervical cancers. In the context of this study, this is due to the limited availability of cancer cell lines harboring other genotypes, but should be considered when validating tests that intend to cover the full spectrum of hrHPV types recommended by the WHO to be covered by *in vitro* diagnostic tests [50]. It is also important to note that the hrHPV DNA or mRNA copies per cell are the number of copies per the total number of cells present in the sample, rather than the number of copies per hrHPV‐infected cells only. Not all cervical squamous epithelial cells are expected to be hrHPV‐infected [30]. Finally, the study is constrained by the evaluation of only 32 samples, which likely do not represent the full biological diversity of cervicovaginal samples and limit the statistical power of the results reported here.

Notably, MS751 had significantly fewer DNA copies per cell than reported in the literature. Recent literature stating the number of HPV 45 copies per cell cites studies from the 1980s that used hybridization to HPV 18 probes and Southern blot analysis, which is only semi‐quantitative [45]. In one study, Yee and colleagues determined that there is ≤ 1 HPV 45 copy per MS751 cell. Geisbill and colleagues confirmed that the hrHPV type was not HPV 18, but HPV 45, and that E6‐E7 mRNA expression was also detectable using Southern and Northern blotting techniques, respectively [51, 52]. In the data presented here, mRNA expression per MS751 cell, while reliably detectable, was also extremely low. While further investigation is needed to determine the cause of these observations, this is, to the authors' knowledge, the only qPCR‐based quantification of HPV 45 DNA copies per cell, and more in‐depth analyses are needed to confirm copy number.

The amount of blood varied among the 32 hrHPV DNA‐positive samples. Samples here were collected into 20 mL of media. Samples collected into a smaller volume will have a higher hemoglobin concentration. Other factors that affect blood content include the type of collection swab used, other procedures performed during the pelvic exam that may promote cervical bleeding, and the patient's menstruation.

The discrepancy in the Partial Integration sample in the amount of plasmid DNA detected following extraction, Exonuclease V treatment, and amplification is likely due to limitations of the modified DNA extraction methods to fully extract cell‐free DNA. In patient samples, viral DNA, even if not integrated, would still be inside the host cell membrane [53]. In the mock samples, circular viral DNA was added to the extracellular media. For this reason, circular viral DNA would not be included in the cell pelleting step, which is the typical first step of DNA extraction from cervicovaginal cells. To account for this, DNA was also extracted from the supernatant according to blood processing instructions for cell‐free nucleic acids; however, the DNA extraction kit is not designed to accommodate PreservCyt media, so a loss in recovery of cell‐free nucleic acids is expected. When constructing mock samples for NAAT development, researchers should consider the intended sample preparation process(es). For example, in a point‐of‐care test with extraction‐free sample preparation or an assay that tests supernatant, the location of nucleic acids within or outside of cells might not make a significant difference in their detectability, while a sample preparation process that relies on separation of cells and supernatant would favor nucleic acids contained within cells. The CaSki cell line is known to contain integrated HPV 16 genomes along with extrachromosomal circular virus–human hybrid DNA, making it a potentially helpful cell line to use when trying to mimic a sample with both integrated and episomal DNA [54].

Altogether, characterization of 32 samples demonstrates the biological diversity of clinical samples and informs protocols for methods to recapitulate them for hrHPV NAAT development. Such research must consider not only nucleic acid quantity, but also the conformation of nucleic acids and the sample matrix, which may contain many inhibitors. Hemoglobin was evaluated as a major interferent in the characterization of clinical and mock samples because both inhibit DNA polymerase and quench fluorescence, so it is important to account for its presence when designing point‐of‐care NAATs [41]. In addition to hemoglobin, other factors that might affect the performance of hrHPV NAATs are sample pH, viscosity, and other proteins and microorganisms that might be found in the cervicovaginal microenvironment. Viscosity was not evaluated in this study because our centrifugal DNA and RNA extraction methods minimize the impact of sample viscosity on PCR results, but it is a factor that can impact sample preparation and NAAT efficiency depending on the methods used [29, 55]. A thorough performance evaluation of a novel hrHPV NAAT would also include systematic testing for potential interference by substances that may be present in the reproductive tract, which was not included here because conventional, commercially‐evaluated extraction and amplification methods were used [56]. In our evaluation of a subset of the cervical samples, we found a very small range in pH, likely due to the buffering effect of the PreservCyt medium, but this could vary more significantly in different collection media. While conventional extraction methods should result in elution of the nucleic acid into a neutral‐pH solution, extraction‐free NAATs may be affected more significantly by variations in sample pH or changes in pH resulting from sample preparation methods [29, 57]. Finally, it is important to consider how stable the mock samples remain over time and under different storage or handling conditions. We recommend generating the mock samples immediately prior to test evaluation, because we did not evaluate the effect of factors such as storage temperature, freeze‐thaw cycles, and time in preservative media for their influence on assay performance. Future work should include a systematic evaluation of mock sample integrity under different conditions to provide more complete recommendations regarding how long they can be stored before use.

## Author Contributions

**Emilie Newsham Novak:** conceptualization, methodology, validation, formal analysis, investigation, writing – original draft, writing – review and editing, visualization. **Ariel Ma:** methodology, validation, investigation. **Cannon J. Hansen:** methodology, validation, investigation, writing – original draft, writing – review and editing, visualization. **Zoha Wazir:** investigation, data curation. **Meaghan Bond:** methodology, writing – original draft, writing – review and editing, supervision. **Michael E. Scheurer:** writing – review and editing, supervision. **Ming Guo:** investigation, writing – review and editing. **Mila P. Salcedo:** resources, writing – review and editing, supervision, project administration. **KMichael E. Scheurer:** conceptualization, resources, writing – review and editing, supervision, project administration. **Rebecca R. Richards‐Kortum:** conceptualization, writing – original draft, writing – review and editing, visualization, supervision, project administration, funding acquisition.

## Conflicts of Interest

The authors declare no conflicts of interest.

## Supporting information

Supporting File

## Data Availability

All analyzed data generated during this study are included in this article and its supplementary files. Raw data are available from the corresponding author upon reasonable request.
