# Supplementary material for: Mock Samples That Mimic Human Cervicovaginal Samples to Accelerate the Development and Evaluation of Assays for High‐Risk HPV for Cervical Cancer Screening
Source: J Med Virol. 2026 Apr 16;98(4):e70931. doi: 10.1002/jmv.70931 (PMC13085960; doi:10.1002/jmv.70931)
Supplement: Supplementary file 1 — Supporting File [file JMV-98-e70931-s001.docx]

**Electronic Supplementary Information**

**Table S1:** Selected commercially available tests for hrHPV.

| **Test (Manufacturer)** | **Target** | **Limit of detection** | **Internal cellular control? (Target)** |
| --- | --- | --- | --- |
| cobas (Roche) | DNA | 150 to 2,400 copies per mL | Yes (β-globin*)* |
| Onclarity (BD) | DNA | 251 to 2,392 copies per mL | Yes (β-globin*)* |
| RealTime (Abbott) | DNA | 500 copies per mL | Yes (β-globin*)* |
| Xpert HPV (Cepheid) | DNA | 2,903 to 50,493 copies per mL | Yes (hydroxymethylbilane synthase (HMBS) |
| Aptima (Hologic) | mRNA | 60 to 1220 copies per mL | No |
| HPV-Proofer (PreTect) | mRNA | 4,000 to 5,000 copies per mL | Yes (U1 small nuclear ribonucleo-protein-specific mRNA.) |

**Table S2:** HPV limit of detection for each NAAT used to evaluate clinical samples.

| **Test** | **Target** | **HPV 16** | **HPV 18** | **HPV 45** |
| --- | --- | --- | --- | --- |
| cobas 4800 | DNA plasmids | 600 copies/mL | 600 copies/mL | 150 copies/mL |
| Xpert HPV | DNA plasmids | 10 copies/reaction | 10 copies/reaction | 10 copies/reaction |
| Aptima HPV | *In vitro* mRNA transcripts | 48.7 copies/reaction | 80.9 copies/reaction | 33.8 copies/reaction |
| qPCR | DNA gBlocks | 10 copies/µL | 10 copies/µL | 10 copies/µL |
| RT-qPCR | Total extracted cellular RNA | 10 copies/µL | 10 copies/µL | 10 copies/µL |

**Table S3:** RT-qPCR plate layout for quantification of HPV 18 and HPV 45 mRNA in Sample 12. Green wells are the HPV 18 RT-qPCR assay. Purple samples are the HPV 45 RT-qPCR assay. Yellow samples are the β-actin RT-qPCR assay. gB = gBlock with the corresponding copy number in copies per µL. The number under each of the yet-unquantified RNA samples is the dilution factor from the stock extracted RNA. Bold wells contain reverse transcriptase; unbold wells do not (no-RT control).

|  | 1 | 2 | 3 | 4 | 5 | 6 | 7 | 8 | 9 | 10 | 11 | 12 |
| --- | --- | --- | --- | --- | --- | --- | --- | --- | --- | --- | --- | --- |
| **A** | **Sample 12** | **Sample 12** | **Sample 12** |  | **18 RNA -1** | **18 RNA -1** | **18 RNA -2** | **18 RNA -2** | **18 RNA -3** | **18 RNA -3** | 18 RNA -1 | 18 RNA -1 |
| **B** | **Sample 12** | **Sample 12** | **Sample 12** |  | **45 RNA -1** | **45 RNA -1** | **45 RNA -2** | **45 RNA -2** | **45 RNA -3** | **45 RNA -3** | 45 RNA -1 | 45 RNA -1 |
| **C** | **Sample 12** | **Sample 12** | **Sample 12** |  |  |  |  |  |  |  |  |  |
| **D** |  |  |  |  |  |  |  |  |  |  |  |  |
| **E** | **NTC** | **NTC** | **18 gB 1e5** | **18 gb 1e5** | **18 gb 1e4** | **18 gb 1e4** | **18 1e3** | **18 1e3** | **18 1e2** | **18 1e2** |  |  |
| **F** | **NTC** | **NTC** | **45 gB 1e5** | **45 gB 1e5** | **45 gB 1e4** | **45 gB 1e4** | **45 gB 1e3** | **45 gB 1e3** | **45 gB 1e2** | **45 gB 1e2** |  |  |
| **G** | **NTC** | **NTC** | **gDNA 1e3** | **gDNA 1e3** | **gDNA 1e2** | **gDNA 1e2** | **gDNA 1e1** | **gDNA 1e1** |  |  |  |  |
| **H** |  |  |  |  |  |  |  |  |  |  |  |  |

**Table S4:** Raw Cq values from RT-qPCR run corresponding to samples in Table S3.

**Figure S1:** Standard curve used for interpolation of HPV mRNA copy number in Sample 12. The HPV 45 gBlock standard curve (equation shown on figure) is used to calculate the extracted HPV 45 standard curve, which is used to calculate the HPV 45 mRNA quantity of Sample 12.

**
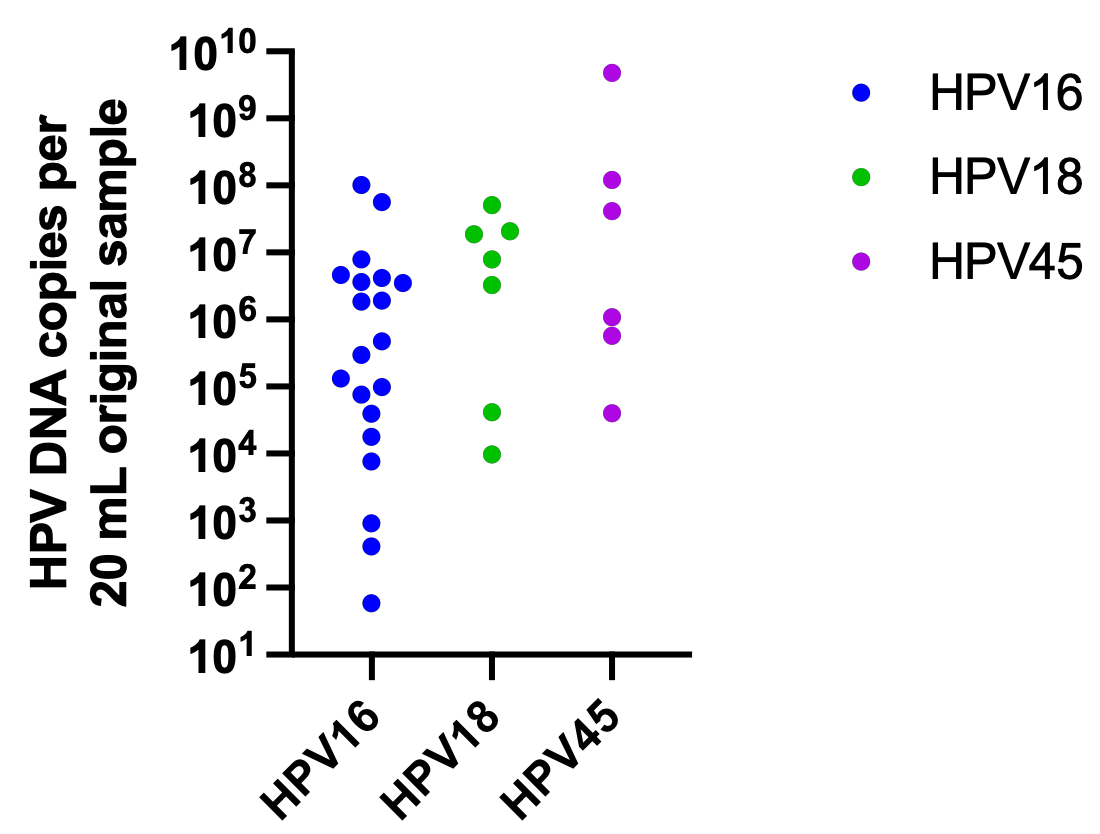
**

**Figure S2:** HPV 16, HPV 18, and HPV 45 DNA copies per 20 mL original PreservCyt sample.

**
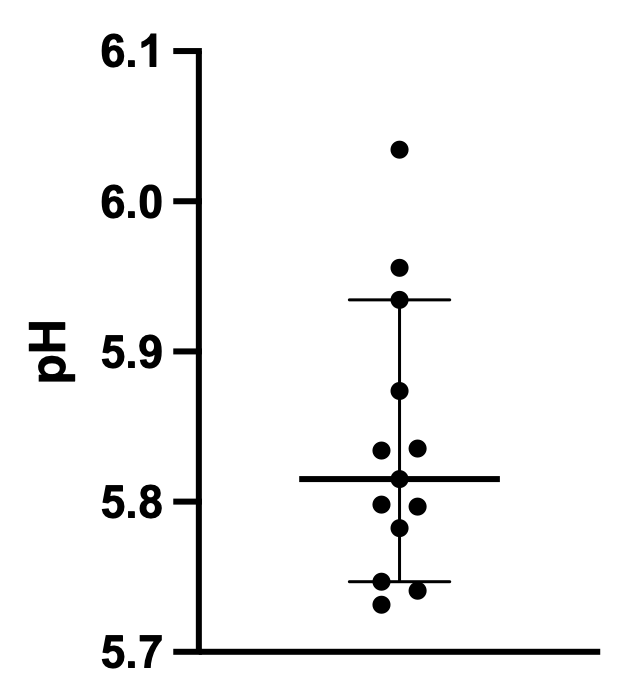
**

**Figure S3:** Mean pH of thirteen HPV-positive cervicovaginal samples. Horizontal lines represent median and interquartile range.

**Table S4:** Xpert HPV results, HPV cycle threshold (Ct) values, and sample adequacy control (SAC) Ct values for the No Integration, Partial Integration, and Full Integration mock samples and the HPV-negative background.

| **Mock Sample** | **GeneXpert Result** | **HPV Ct** | **SAC Ct** |
| --- | --- | --- | --- |
| No Integration | HPV 18/45 | 30.8 | 31.8 |
| Partial Integration | HPV 18/45 | 22.4 | 26.2 |
| Full Integration | HPV 16 | 32.3 | 31.7 |
| Background | NEG | N/A | 32.5 |

**Table S5:** Hemoglobin concentration of mock samples.

| **Mock sample** | **Hemoglobin concentration (g/dL)** |
| --- | --- |
| Full Integration (SiHa + Pooled Neg.) | 0.00111471 |
| No Integration (HPV45 Plasmid + pooled neg.) | 0.00116747 |
| Partial Integration (HeLa + Pooled Neg. + HPV18 plasmid) | 0.0012 |
| Pooled negative | 0.00118073 |

**Table S6:** Results summary of all clinical samples tested. “N/A” indicates that the given result is not available.

| **Sample number** | **HPV genotype** | **HPV DNA copies/**  **cell** | **Percent circular (non-integrated) DNA** | **HPV mRNA copies/**  **cell** | **Hemoglobin concentration (mg/dL)** | **Pathology of highest-grade tissue specimen** |
| --- | --- | --- | --- | --- | --- | --- |
| 1 | 16 | 2.319 | 8.6% | 0.00001889 | 0.07038 | VAIN 2/3 |
| 2 | 45 | 0.004326 | 100% | 0 | 0.5640 | N/A |
| 3 | 16 | 0.3217 | 22.99% | 0 | 1.217 | N/A |
| 4 | 18 | 0.001101 | 0% | 0 | 0.5399 | N/A |
| 5 | 16 | 0.008503 | 4.95% | 0.005857 | 28.21 | CIN 2 |
| 6 | 16 | 0.001245 | 100% | 0 | 0.2634 | N/A |
| 7 | 16 | 0.02199 | 8.54% | 0 | 0.2100 | N/A |
| 8 | 16 | 0.5087 | 57.92% | 0.005376 | 5.727 | CIN 2/3 |
| 9 | 18 | 185.7 | 68.11% | 0 | 5.580 | N/A |
| 10 | 16 | 0.0002 | 0% | 0 | 0.2564 | N/A |
| 11 | 45 | 23.29 | 26.07% | 0 | 0.3982 | CIN 1 |
| 12 | 45 | 1315 | 0.58% | 2903 | 0.5798 | Adenocarcinoma |
| 13 | 16 | 2.263 | 15.39% | 0 | 1.736 | ASC |
| 14 | 16 | 0.1304 | 18.85% | 0 | 3.558 | N/A |
| 15 | 16 | 2.689 | 16.80% | 0.004118 | 1.162 | VIN 2/3 |
| 16 | 16 | 4.706 | 26.47% | 1.522 | 0.2294 | AIS |
| 17 | 18 | 11.34 | 24.45% | 0 | 0.3846 | N/A |
| 18 | 16 | 36.47 | 17.32% | 0 | 0.5386 | N/A |
| 19 | 18 | 21.34 | 6.00% | 0 | 0.1847 | VAIN 2 |
| 20 | 16 | 9.742 | 50.18% | 0 | 1.504 | N/A |
| 21 | 18 | 0.02567 | 0% | 0 | 6.449 | Benign/  normal |
| 22 | 16 | 0.1382 | 0% | 0 | 5.385 | CIN 1 |
| 23 | 16 | 87.65 | 1.16% | 0 | 0.03444 | Benign/  normal |
| 24 | 18 | 15.19 | 0% | 4.120 | 2.205 | CIN 2/3 |
| 25 | 16 | 0.7016 | 0% | 0 | 0.2829 | VIN 3 |
| 26 | 16 | 4.347 | 0% | 0 | 1.084 | Benign/  normal |
| 27 | 16 | 0.01707 | 0% | N/A | 1.074 | CIN 3 |
| 28 | 45 | 18620 | 1.89% | 0.1091 | 0 | CIN 1 |
| 29 | 45 | 5.374 | 0% | 0.05703 | 0.4607 | Benign/  normal |
| 30 | 16 | 6.870 | 0.14% | N/A | 0.4833 | CIN 3 |
| 31 | 18 | 33450 | 3.15% | 28.16 | 0.1061 | CIN 2 |
| 32 | 16 | 56.98 | 0.71% | 0.02313 | 1.211 | CIN 3 |
